# Construction and Clinical Relevance of a Predictive Model of Coronary Microcirculatory Dysfunction in Patients With Acute Myocardial Infarction Following Percutaneous Coronary Intervention

**DOI:** 10.31083/RCM38533

**Published:** 2025-06-30

**Authors:** Shuai Wang, Yuanyuan Zhao, Yanlong Zhao, Yanling Wang, Zhenxing Fan, Zhi Liu

**Affiliations:** ^1^Department of Emergency, Xuanwu Hospital, Capital Medical University, 100053 Beijing, China; ^2^Department of Cardiology, Xuanwu Hospital, Capital Medical University, 100053 Beijing, China; ^3^Department of Geriatrics, Xuanwu Hospital Capital Medical University, 100053 Beijing, China

**Keywords:** acute myocardial infarction, angiography-derived microcirculatory resistance, coronary microcirculatory dysfunction, predictive model

## Abstract

**Background:**

Coronary microcirculatory dysfunction (CMD) after percutaneous coronary intervention (PCI) in patients suffering from acute myocardial infarction (AMI) may adversely affect prognosis. The objective of this study was to assess the postoperative microcirculatory status and to construct a predictive model for CMD.

**Methods:**

This study is a retrospective analysis of 187 AMI patients who underwent PCI at Xuanwu Hospital. Patients were divided into two cohorts based on postoperative angiography-derived microcirculatory resistance (AMR) values: a non-CMD group (AMR <250 mmHg*s/m, n = 93) and a CMD group (AMR ≥250 mmHg*s/m, n = 76). Clinical and laboratory data were extracted, predictive models were constructed and risk factors associated with CMD were identified through the implementation of LASSO regression analyses.

**Results:**

The non-CMD group (n = 93) had a significantly lower body mass index (BMI) (25.40 ± 2.84) and a higher proportion of males (91.4%) compared to the non-CMD group (n = 76) (BMI: 26.64 ± 3.74, *p* < 0.05; males: 78.9%, *p* < 0.05). The non-CMD group also exhibited lower Creatine Kinase (CK) levels, glucose levels (GLU), mean platelet volume (MPV), and platelet distribution width (PDW). LASSO regression identified significant predictors of CMD after PCI in AMI patients. A nomogram showed excellent predictive performance (area under curve (AUC): 0.737) and higher net benefit compared to individual models.

**Conclusion:**

The predictive model developed in this study effectively identifies the risk of microcirculatory dysfunction in AMI patients after PCI, providing important insights for clinical decision-making. Future research should further validate the external applicability of this model and explore its potential in clinical practice.

**Clinical Trial Registration:**

NCT06062316, https://clinicaltrials.gov/study/NCT06062316?term=NCT06062316&rank=1, registration time: December 21, 2023.

## 1. Introduction

Acute myocardial infarction (AMI), a critical form of coronary heart disease, poses a grave risk to human life and health and is a leading factor in cardiovascular-related deaths. The most effective treatment for AMI at present is reperfusion therapy, which is predominantly carried out via percutaneous coronary intervention (PCI) [1].

In particular, in more than half of AMI patients who achieve successful PCI, revascularization might not entirely eliminate coronary microcirculatory dysfunction (CMD). Furthermore, CMD is associated with an increased probability of major adverse cardiovascular events (MACEs), irrespective of whether epicardial coronary narrowing is present [2, 3]. Prompt identification of CMD facilitates the implementation of bespoke therapeutic strategies, with the potential to enhance myocardial perfusion and improve clinical outcomes. It is important to emphasize that despite reaching PCI thrombolysis in myocardial infarction (TIMI) grade 3 flow, many patients still have suboptimal tissue perfusion, which subsequently leads to unfavorable prognostic outcomes. This might be linked to causes like ischemia-reperfusion injury, coronary microvascular obstruction (MVO) and inflammatory responses [4].

Impairment of microvascular function significantly contributes to the development of myocardial ischemia, with both prognostic and symptomatic implications [5]. It is essential to understand the mechanisms of CMD and the intracoronary tools available to detect it, as these can help unmask the main underlying mechanisms and guide therapeutic interventions [6].

Cardiovascular magnetic resonance (CMR) is an established technique for the specific identification of CMD and MVO. However, CMR is typically conducted a period of 2 to 7 days post-primary percutaneous coronary intervention (PPCI), which may be too late for timely intervention. Furthermore, CMR is not appropriate for patients with kidney problems, which restricts its use in clinical settings [7, 8].

Recently, the angio-based quantitative flow ratio (QFR) has become a promising substitute for fractional flow reserve (FFR), providing a new approach to streamline coronary functional evaluation without requiring pressure wires or adenosine [9]. The significant role of QFR in forecasting future adverse events in patients who have stable coronary artery disease (CAD) and non-STEMI has been established, highlighting the necessity of immediate physiological evaluation of QFR following the procedure as an essential resource for optimizing PCI [10]. Furthermore, the diagnostic accuracy of a CMD assessment has been shown to be favorable when utilizing angiography-derived microcirculatory resistance (AMR) calculated from QFR without the need for guidewires or adenosine. Thus, this method represents a viable clinical substitute for invasive pressure guidewire assessment of the index of microcirculatory resistance (IMR) [11, 12]. Relevant studies have shown that AMR has high consistency and diagnostic accuracy in predicting IMR [13, 14]. Considering the patient’s condition and the availability of medical resources, the application of the combination of these two approaches can enable a more comprehensive assessment of microcirculation disorders [15, 16].

Given the non-invasive and instantaneous nature of QFR and AMR, the objective of this study was to determine the clinical significance of conducting an immediate angiography-based evaluation of coronary function in assessing risk levels among AMI patients undergoing PCI with TIMI grade 3 flow. Our objective is to leverage biomarker and clinical multimodal data to create a prognostic scoring system for CMD in AMI patients undergoing PCI. This model may facilitate earlier detection of microvascular impairment and guide therapeutic decisions.

## 2. Materials and Methods

### 2.1 Study Design and Population

**Fig. 1.**
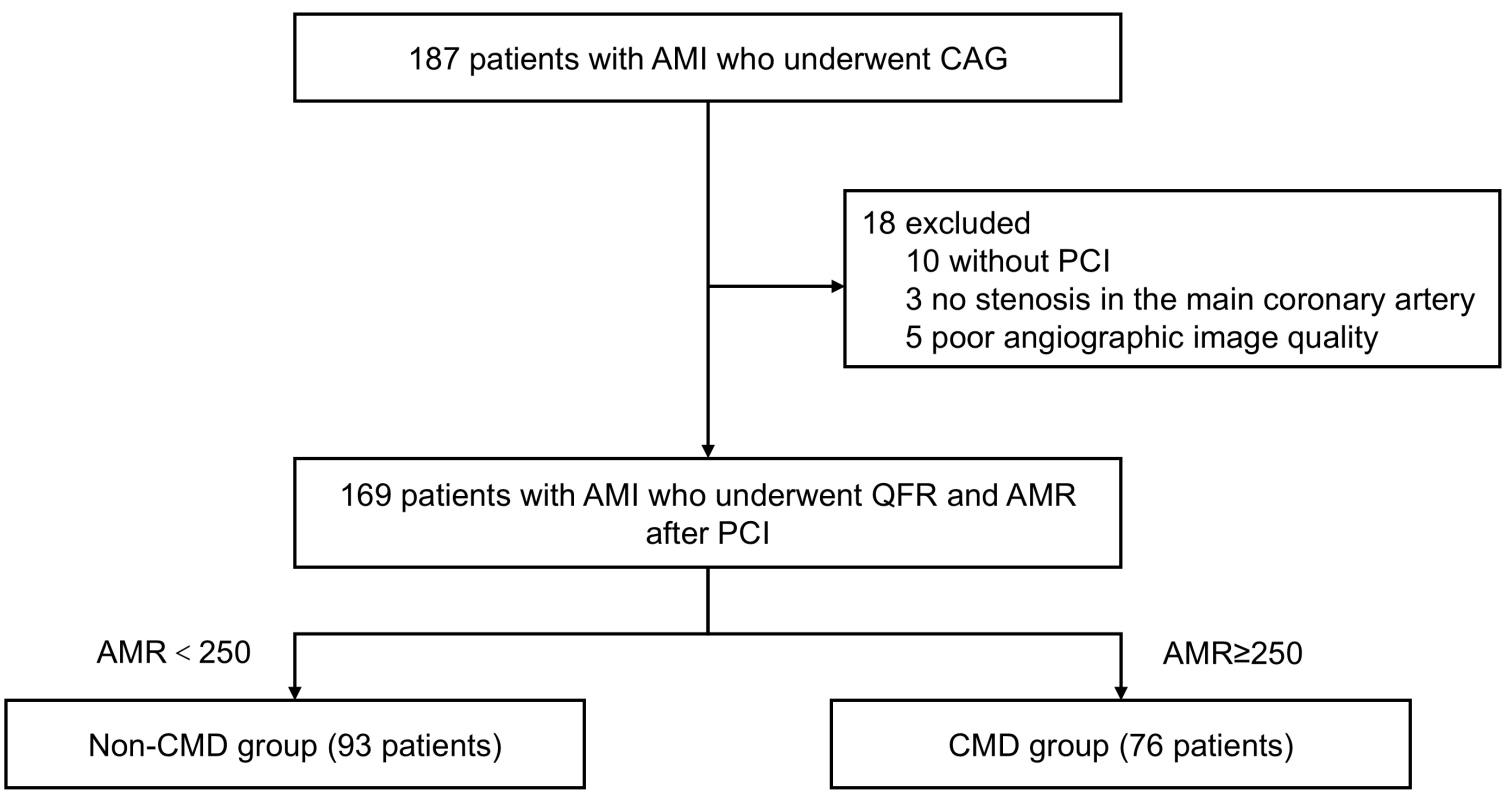
**Study flowchart diagram**. AMI, acute myocardial infarction; AMR, angio-derived microcirculatory resistance; CAG, coronary angiography; CMD, coronary microvascular dysfunction; PCI, percutaneous coronary intervention; QFR, quantitative flow ratio.

The study retrospectively analyzed consecutive patients aged 18 and above with AMI who underwent PCI after experiencing symptoms onset at Xuanwu Hospital, Capital Medical University. The criteria used to diagnose AMI were formulated according to the most recent guidelines provided for acute coronary syndromes (ACSs) [1]. The study encompassed 187 AMI patients who were subjected to coronary angiography (CAG). Among the initially included patients, 18 were excluded for the following reasons: 10 patients did not undergo PCI, 3 patients had no stenosis in the main coronary artery, and 5 patients had poor angiographic image quality. After exclusions, 169 patients with AMI who had QFR and AMR evaluations following PCI were included in the study. Considering a postoperative AMR level of 250 mmHg*s/m (as there is no defined threshold for AMR in CMD, recent study data were used [17]). According to AMR values, patients were divided into two groups: the non-coronary microcirculation dysfunction group (93 patients, AMR <250) and the coronary microcirculation dysfunction group (76 patients, AMR ≥250) (Fig. 1).

The study follows the ethical framework defined in the Declaration of Helsinki. The present study was an observational, single-center clinical trial (ClinicalTrials.gov number, NCT06062316) and was approved by the Ethics Committee of the Xuanwu Hospital Capital Medical University. Written informed consent was obtained from all participants.

### 2.2 Laboratory Tests

The following parameters were retrospectively extracted from medical records: age, gender, smoking and alcohol status, culprit vessel, and clinical comorbidities such as hypertension or diabetes mellitus. Serum biochemical markers, such as markers of myocardial injury, glucose, blood lipids, serum creatinine, and blood routine were measured in the hospital’s clinical laboratory using standard automated methods. Serum inflammatory markers were measured using these ratios: neutrophil-to-lymphocyte ratio (NLR) is calculated by dividing the neutrophil count by the lymphocyte count, and platelet-to-lymphocyte ratio (PLR) is determined by dividing the platelet count by the lymphocyte count; the Systemic Inflammation Index (SII) is calculated by multiplying platelets and neutrophils, then dividing by the lymphocyte count [18, 19].

### 2.3 Coronary Physiology Analysis

**Fig. 2.**
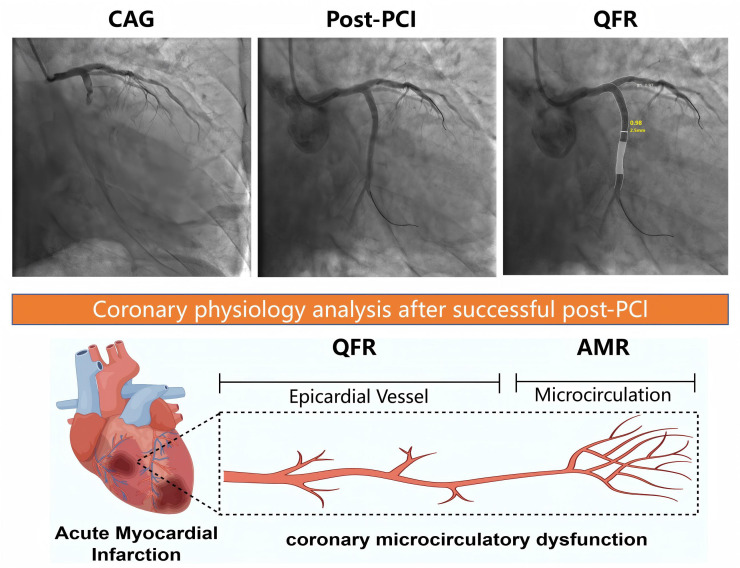
**Simulation of Coronary physiology analysis**.

The vessel responsible was identified based on two main criteria: firstly, it was established by correlating the angiographic findings with the existence of plaque instability or thrombus. Secondly, both electrocardiographic and echocardiographic results were considered. Furthermore, two angiographic images were obtained, ensuring a minimum separation of 25° in their projection angles. The images received evaluation and validation from two seasoned interventional cardiologists. Following this, The AngioPlus Pro system, created by Pulse Medical Technology in Shanghai, China, employs artificial intelligence to conduct QFR and AMR calculations, receiving angiographic images for analysis [9]. For QFR assessment, the system has been developed to pinpoint the best post-PPCI projections that minimize vessel foreshortening and overlap. The system first identifies proximal and distal anatomical landmarks, after which vessel contours are automatically detected, with manual adjustments made when necessary. The incorporation of significant side branches that have a diameter of at least 1.0 mm guarantees precise QFR measurements. In addition to QFR, the AngioPlus Pro system automatically calculates parameters such as the AMR (Fig. 2). A certified analyst, who was not informed of the clinical data, performed all the coronary physiology measurements. Briefly, the investigation recruited PCI-treated AMI patients, employing AMR for non-invasive microcirculatory evaluation of the culprit artery. Both AMR and QFR values were recorded following the intervention.

## 3. Statistical Analysis

Continuous variables were expressed as mean ± standard deviation (SD) or median (IQR), while categorical variables were reported as counts (percentages). For categorical variables, we used chi-square test or Fisher’s exact test; For continuous variables, *t*-test or Mann-Whitney U test were used based on the distribution of the data. Considering the collinearity among the collected variables, LASSO regression analysis was used to screen the predictors. Multiple factors influencing CMD were evaluated by constructing a nomogram model. The model’s discriminatory performance was assessed using receiver operating characteristic (ROC) curve analysis, where the area under curve (AUC) acted as a quantitative indicator of predictive accuracy. A *p*-value < 0.05 was considered significant. All analyses were performed using R Statistical Software (Version 4.2.2, http://www.R-project.org, The R Foundation) and Empower Stats (http://www.empowerstats.com, X&Y Solutions Inc., Boston, MA, USA).

## 4. Results

### 4.1 Clinical Features of the Study Population

The following table presents a comparison of clinical and laboratory parameters between two groups based on a threshold of 250 mmHg*s/m. The group with values less than 250 mmHg*s/m (n = 93) exhibited a significantly lower body mass index (BMI) (25.40 ± 2.84) in comparison to the group with values equal to or greater than 250 mmHg*s/m (n = 76) (26.64 ± 3.74, *p* = 0.015). A higher proportion of males was observed in the lower group (91.4% vs. 78.9%, *p* = 0.021). While age, smoking status, alcohol consumption, incident hypertension, and diabetes did not demonstrate significant differences. Laboratory results indicated that creatine kinase (CK) levels were significantly elevated in the higher group (624.00 [214.00, 1406.00] vs. 1039.50 [366.5, 2696.00]. In addition, glucose levels were found to be higher in the higher group (6.71 ± 3.21 vs. 7.71 ± 3.19, *p* < 0.05). Furthermore, mean platelet volume (MPV) and platelet distribution width (PDW) were found to be significantly lower in the lower group (10.42 ± 0.90 vs. 10.01 ± 0.80, *p* < 0.05; 12.01 ± 1.91 vs. 11.07 ± 1.80, *p* < 0.05). The research indicates that notable variations exist in both clinical and laboratory parameters linked to a cutoff of 250 mmHg*s/m (all *p* < 0.05, Table 1).

**Table 1.** **Baseline data comparison for patients with or without CMD**.

Variables		non-CMD <250 mmHg*s/m	CMD ≥250 mmHg*s/m	*p*
N		0 (n = 93)	1 (n = 76)	
BMI, kg/m^2^		25.40 ± 2.84	26.64 ± 3.74	0.015
Age, yrs		57.81 ± 11.56	59.01 ± 12.38	0.514
Male sex, n (%)		85 (91.4)	60 (78.9)	0.021
Smoking, n (%)		63 (67.7)	43 (56.6)	0.135
Alcohol, n (%)		71 (76.3)	55 (72.4)	0.555
Incident HTN, n (%)		43 (46.2)	39 (51.3)	0.511
Incident DM, n (%)		21 (22.6)	19 (25)	0.713
Culprit vessel, n (%)				0.074
	LAD	50 (53.8)	36 (47.4)	
	LCX	9 (9.7)	17 (22.4)	
	RCA	34 (36.6)	23 (30.3)	
Hs-TnI, ng/L		10.50 (2.44, 33.30)	15.00 (3.01, 38.80)	0.228
CK-MB, IU/L		101.00 (25.00, 242.00)	153.00 (34.30, 448.75)	0.130
MYO, µg/L		74.65 (31.85, 189.75)	87.30 (41.45, 442.25)	0.153
CK, IU/L		624.00 (214.00, 1406.00)	1039.50 (366.50, 2696.00)	0.010
SCR, µmol/L		72.61 ± 16.45	68.15 ± 15.33	0.072
HDL-C, mmol/L		1.08 ± 0.30	1.09 ± 0.25	0.696
LDL-C, mmol/L		2.87 ± 1.10	2.98 ± 0.97	0.495
GLU, mmol/L		6.71 ± 3.21	7.71 ± 3.19	0.002
NLR		5.16 (2.70, 7.29)	5.62 (3.79, 8.84)	0.087
PLR		154.05 (109.89, 202.70)	172.08 (107.54, 234.81)	0.247
SII		1052.17 (636.36, 1854.00)	1465.84 (726.01, 2273.38)	0.171
PCT		0.25 ± 0.10	0.23 ± 0.06	0.350
MPV		10.42 ± 0.90	10.01 ± 0.80	0.003
PDW		12.01 ± 1.91	11.07 ± 1.80	0.001

BMI, body mass index; CK, creatine kinase; CK-MB, creatine kinase-MB; DM, diabetes mellitus; GLU, glucose; HDL-C, high-density lipoprotein cholesterol; Hs-TnI, high-sensitivity troponin I; HTN, hypertension; LDL-C, low-density lipoprotein cholesterol; LAD, left anterior descending artery; LCX, left circumflex artery; MPV, mean platelet volume; MYO, myoglobin; NLR, neutrophil-to-lymphocyte ratio; PCT, plateletcrit; PLR, platelet-to-lymphocyte ratio; PDW, platelet distribution width; RCA, right coronary artery; SII, systemic immune-inflammation index; SCR, serum creatinine.

### 4.2 Construction of Prediction Nomogram

**Fig. 3.**
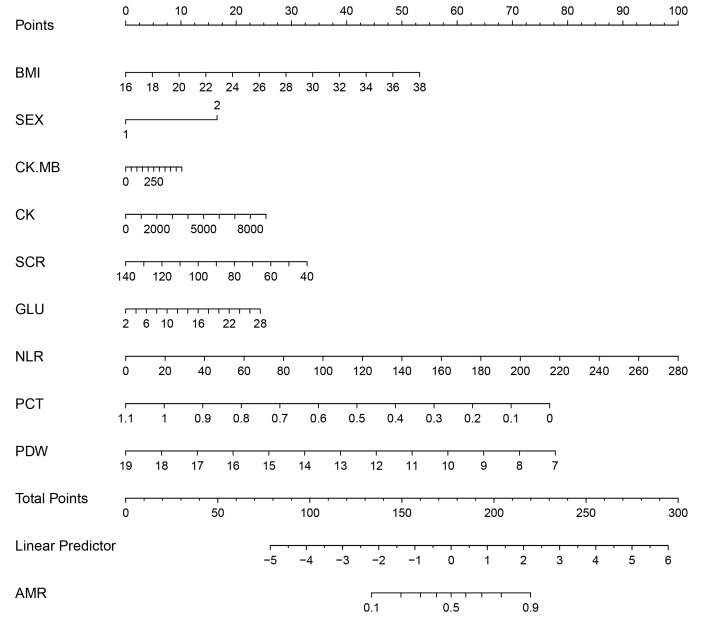
**A prediction nomogram was formulated from the ideal multivariate logistic regression model to predict CMD probability**.

All variables underwent dimensionality reduction via LASSO regression (Table 1). A procedure based on 10-fold cross-validation identified the optimal λ parameter, chosen based on the point of minimal cross-validation error for subsequent modeling. Subsequently, the number of variables with non-zero regression coefficients at this stage was determined. The analysis using LASSO regression highlighted certain variables—BMI, sex, creatine kinase-MB (CK-MB), CK, serum creatinine (SCR), glucose levels (GLU), NLR, plateletcrit (PCT), and PDW—as significant predictors of CMD occurrence following PCI in patients with AMI (**Supplementary Figs. 1,2**). A nomogram was created utilizing routine medical factors (Fig. 3).

### 4.3 Performance of Prediction Nomogram

**Fig. 4.**
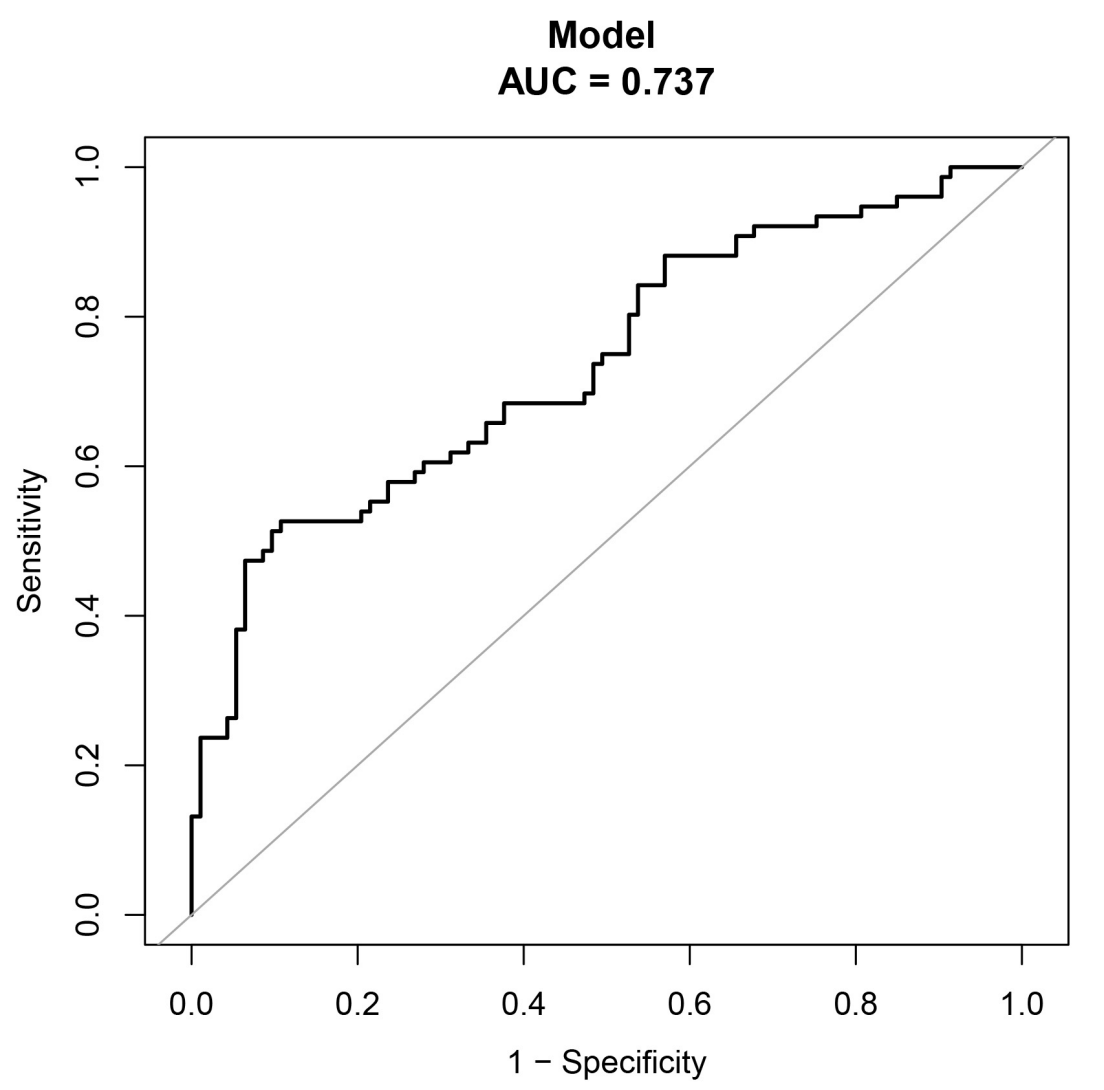
**The predictive efficacy of the nomogram in predicting CMD was validated through receiver operating characteristic (ROC) curve analysis**.

To guarantee the robustness and reliability of the predictive model, we performed Bootstrap resampling on the original sample (169 patients), and each resample was repeated 500 times with the same sample size as the original sample (169 patients). This approach is effective in assessing the variability and confidence of model predictions. ROC analyses are performed by this method and the results show that the constructed nomograms exhibit excellent discriminatory performance in predicting CMD. The AUC of 0.737 (95% confidence interval: 0.66–0.81) reflects the model’s efficacy in this regard (Fig. 4).

The decision curve analysis (DCA) curve shows the clinical benefits of the model and the threshold for the best applicability. The DCA shows the overall clinical benefits predicted by using this model. The results show that the nomogram of this model has certain clinical practicability (**Supplementary Fig. 3**).

## 5. Discussion

This study presents a novel approach to assessing CMD in AMI patients following PCI by utilizing postoperative AMR values. Notably, BMI, sex, CK-MB, CK, SCR, GLU, NLR, PCT, and PDW emerged as significant risk factors. These findings align with existing literature that highlights the role of metabolic and hemodynamic parameters in influencing microcirculatory health.

Several studies have indicated that BMI and glucose levels have a detrimental impact on microcirculatory function [20, 21]. Elevated blood glucose levels have been associated with endothelial dysfunction, which may contribute to impaired microcirculation in AMI patients.

The presence of obesity-induced chronic low-grade inflammation has been demonstrated to play a significant role in the development of atherosclerosis, particularly concerning visceral fat. Elevated levels of oxidative stress in obese patients are accompanied by an inflammatory response, and these factors can further compromise coronary microcirculation. This current research has shown a positive relationship between a higher BMI and the likelihood of experiencing coronary microcirculatory disorders [22, 23].

In the case of AMI, levels of CK-MB were found to be significantly elevated, and a positive correlation was observed between these levels and the extent of the myocardial infarction (MI) [24]. It is hypothesized that this may be related to insufficient myocardial blood supply, due to impaired coronary microcirculation [25]. Although CK-MB or CK is a sensitive indicator of myocardial injury, its level may be affected by several factors. Elevated creatinine levels may indirectly affect coronary microcirculation by affecting renal function and systemic inflammatory status. Changes in serum creatinine levels may be associated with impaired coronary microvascular vasodilatory capacity [26].

Elevated levels of NLR, a marker of inflammation, are strongly associated with the severity and complexity of CAD and microcirculatory dysfunction. The study noted that NLR is significantly associated with the risk of CAD and ACS and that its elevation is associated with an increased incidence of cardiovascular events [27, 28]. Thus, elevated NLR may reflect this inflammatory state and thus be an important biomarker for assessing coronary microcirculatory disorders.

Coronary microcirculatory impairment is defined as impaired coronary microvascular function, and this impairment is closely related to platelet activation and aggregation. Platelets play an important role in the inflammatory response and vascular endothelial damage. The findings of the present study demonstrated that PCT and PDW could serve as screening variables for the prediction of CMD. A study has indicated that PDW can be utilized as a preliminary test to identify high-risk patients for myocardial infarction, thus underscoring its potential utility in clinical settings for risk stratification [29]. Specifically, one study demonstrated that PDW is an independent predictor of adverse outcomes in patients with ACS [29]. This underscores the importance of PDW in identifying patients who may benefit from more aggressive antiplatelet therapy during MI events [30]. Moreover, PDW has been identified as an independent predictor of mortality in patients with cardiovascular conditions, further emphasizing its relevance in the prognosis of MI patients. Research has demonstrated that elevated PDW levels are associated with increased mortality rates in various cardiovascular contexts, including ACSs and post-MI scenarios [31]. For instance, a study revealed that patients with elevated PDW values exhibited significantly higher mortality rates compared to those with lower values [31]. The multifaceted role of PDW in both risk stratification and prognostic assessment underscores its potential as a valuable tool in clinical practice for managing patients at risk of MI and related complications. PCT is positively correlated with other platelet parameters such as PDW and PLR, all of which are associated with the development of microcirculatory disorders [32, 33]. The PCT serves as a crucial indicator for the long-term outlook and microcirculatory impairment in individuals experiencing ACSs. PCT was found to be negatively correlated with microcirculatory parameters such as coronary blood flow velocity and left anterior descending (LAD) diastolic flow time (DDT), suggesting that PCT could potentially be used as a biomarker to assess dysfunction in coronary microcirculation [34].

Research has demonstrated that myocardial microvascular obstruction is a significant predictor of left ventricular remodeling and heart failure events, and the presence of CMD continues to augment the risk of heart failure following complete and timely revascularisation [19, 35, 36].

The occurrence of CMD is closely related to a variety of clinical and biological factors and has an important impact on the prognosis of patients. By constructing prediction models based on multiple variables, high-risk patients can be effectively identified, thus providing important support for early intervention and improved prognosis [37, 38]. In patients with AMI, CMD may adversely affect prognosis after PCI. The occurrence of CMD is related to many factors. For example, inflammatory biomarkers, sex differences, NLR, diabetes and Gensini Scores were discovered to independently predict post-PCI CMD in AMI patients [39, 40, 41]. A study has shown that there is a significant correlation between a hypercoagulable state and CMD, and a hypercoagulable state significantly increases the risk of CMD [42].

The study has an innovative approach to categorizing the microcirculatory status of AMI patients after PCI based on postoperative AMR values. By employing LASSO regression and multivariate logistic regression analyses, the study identified key predictors of CMD, including BMI, QFR, GLU, PDW, and PCT. The predictive model developed from these variables demonstrated strong discriminatory ability, achieving an AUC of 0.737 during internal validation. This level of predictive accuracy is promising and suggests that the model could serve as a valuable tool for clinicians in the early identification of patients at risk for microcirculatory dysfunction. Early intervention in these patients may improve clinical outcomes, as CMD has been linked to adverse prognoses in AMI.

Moreover, CMD is not only a predictor of adverse outcomes but also reflects underlying pathophysiological changes that occur during ischemic events. The relationship between CMD and myocardial ischemia underscores the importance of assessing microvascular integrity in patients with AMI [43, 44]. This model provides valuable insights for the early identification and intervention of CMD in AMI patients, emphasizing the significance of microcirculatory dysfunction in prognosis and offering a new perspective for optimizing postoperative management strategies following PCI.

Some study limitations merit discussion. First, the retrospective collection of data from electronic health records inherently predisposes to possible selection and information biases. Second, these results should be interpreted prudently, and future validation through prospective randomized controlled trials is required. Finally, despite using internal validation techniques to test the model’s predictive capability at this stage, external validation is considered the gold standard for ensuring dataset validity.

## 6. Conclusion

In conclusion, this study contributes to the growing body of evidence regarding the importance of microcirculatory status in AMI patients post-PCI. The identification of key predictors and the development of a predictive model provide a foundation for future research and clinical applications aimed at optimizing patient care and improving prognostic outcomes in this high-risk population.

## Availability of Data and Materials

The datasets used and analyzed during the current study are available from the corresponding author on reasonable request.

## Supplementary Material

Supplementary material associated with this article can be found, in the online version, at https://doi.org/10.31083/RCM38533.
