# Supplementary material for: Construction and Clinical Relevance of a Predictive Model of Coronary Microcirculatory Dysfunction in Patients With Acute Myocardial Infarction Following Percutaneous Coronary Intervention
Source: Rev Cardiovasc Med. 2025 Jun 30;26(6):38533. doi: 10.31083/RCM38533 (PMC12230841; doi:10.31083/RCM38533)
Supplement: Supplementary file 1 [file 2153-8174-26-6-38533-s1.docx]

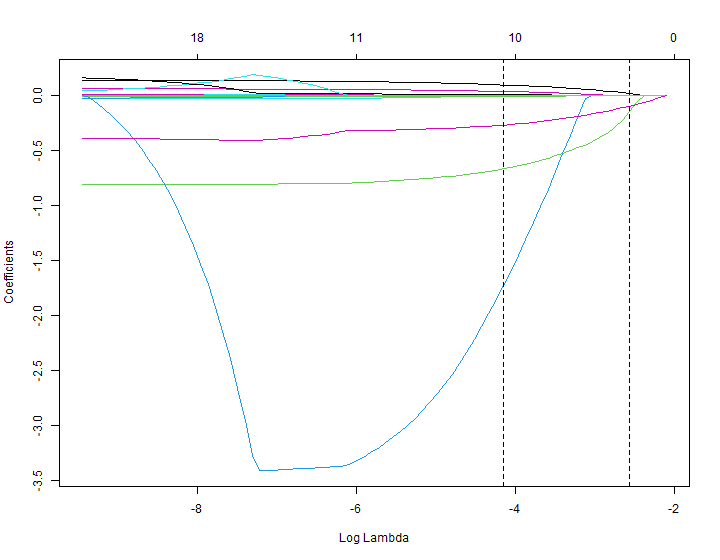
**Supplemental Figure 1**. The least absolute shrinkage and selection operator (LASSO) regression model is a statistical technique that can be used to identify the trendlines of model coefficients for clinical variables that may be associated with the occurrence of coronary microvascular dysfunction (CMD) in patients. Based on lambda.min, the most significant influencing CMD regression equation is screened out as logit (*CMD*) = 0.09342*BMI-0.66872*SEX+8e-04*CK.MB+0.00012*CK-0.01114*SCR+ 0.03463*GLU+0.00549*NLR-1.72567*PCT-0.26959*PDW.


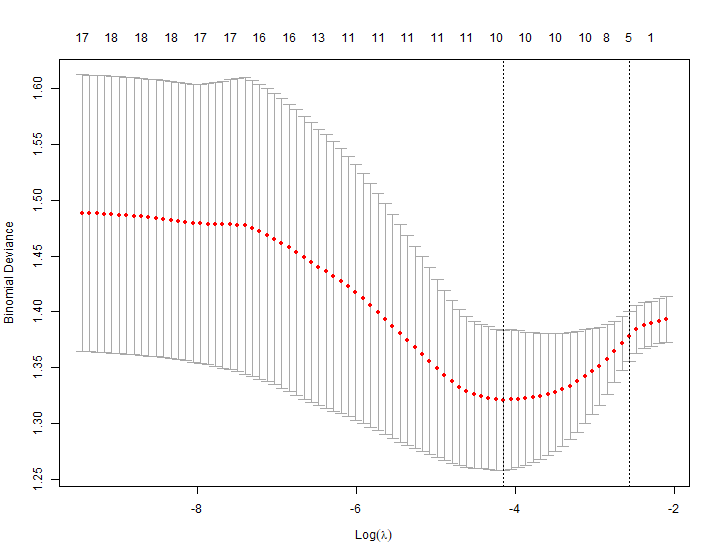


**Supplemental Figure 2**. The selection of the tuning parameter (λ) was conducted using a cross-validation error curve derived from a 10-fold cross-validation approach. Optimal values were determined based on the criteria of the minimum error and one standard error, as indicated by the vertical lines on the curve.


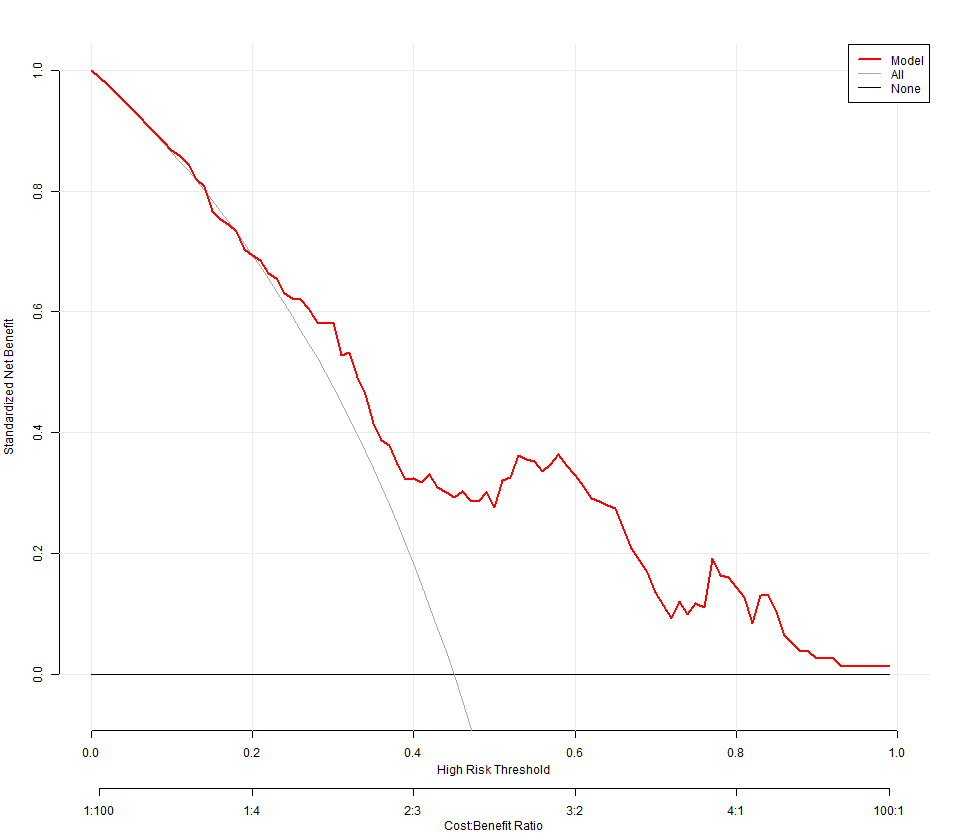


**Supplemental Figure 3**. Net benefit rate of the prediction nomogram and separate models for the independent variables.
